# Improvement in pulmonary vascular resistance with IMPELLA 5.5 as a bridge to orthotopic heart transplantation

**DOI:** 10.3389/fcvm.2026.1935715

**Published:** 2026-10-20

**Authors:** Jackson Rankin, Bartlomiej Imielski, Allyson Howard-Green, Anju Saxena, Deevya Raman, Austin Seals, Kenneth S. Paik, Priyesh A. Patel, Joseph Mishkin, Hassan Khan, Aderonke O. Adeniyi, Adrian Lata, Olivia Gilbert

**Affiliations:** 1 Department of Internal Medicine, Wake Forest University School of Medicine, Atrium Health Wake Forest Baptist, Winston-Salem, NC, United States; 2 Department of Cardiothoracic Surgery, Wake Forest University School of Medicine, Atrium Health Wake Forest Baptist, Winston-Salem, NC, United States; 3 Wake Forest University School of Medicine, Winston-Salem, NC, United States; 4 Department of Cardiovascular Medicine, Wake Forest University School of Medicine, Atrium Health Wake Forest Baptist, Winston-Salem, NC, United States; 5 Department of Cardiovascular Medicine, Wake Forest University School of Medicine- Charlotte Campus, Sanger Heart and Vascular Institute, Atrium Health, Charlotte, NC, United States

**Keywords:** heart transplant, impella 5.5, pulmonary hypertension due to left heart disease, pulmonary vascular resistance (PVR), temporary mechanical circulatory support

## Abstract

**Purpose:**

The Impella 5.5 is a temporary micro-axial mechanical circulatory support (tMCS) device used for left ventricular (LV) support and unloading as a bridge to advanced therapies. However, the short-term impact of LV unloading on pulmonary hypertension (PH) has not been clearly defined. This study explored whether Impella 5.5 support results in a reduction in PH in patients bridged to orthotopic heart transplantation (OHT).

**Methods:**

This retrospective observational study examined 75 patients from two tertiary cardiac centers who had an Impella 5.5 placed during an admission (2018–2025) that culminated in OHT. Right heart catheterization (RHC) and transthoracic echocardiography (TTE) data were collected, including studies obtained before and after Impella placement. The primary outcomes were changes in pulmonary vascular resistance (PVR), mean pulmonary artery pressure (mPAP), and pulmonary artery systolic pressure (PASP). Secondary outcomes included changes in qualitative right ventricular (RV) systolic function, transpulmonary gradient (TPG), and pulmonary artery pulsatility index (PAPi). Paired and unpaired analyses were conducted. For the primary unpaired analysis, differences between the pre-Impella and during-Impella time points were assessed using the Wilcoxon rank-sum test for continuous variables and chi-square or Fisher's exact test for categorical variables.

**Results:**

Following Impella 5.5 placement, median PVR by Fick and Thermodilution decreased from 2.8 (IQR, 2.1–3.8) to 1.5 (1.2–2.1) Wood Units (WU) (*p* < 0.001) and from 2.4 (1.9–2.8) to 1.3 (1.0–1.5) WU (*p* < 0.001), respectively. Median mPAP and PASP decreased from 35.5 (31.0–41.8) to 24.5 (20.2–32.0) mmHg (*p* < 0.001) and from 48 (42.0–55.8) to 37.5 (27.5–42.0) mmHg (*p* < 0.001), respectively. Secondary outcomes showed an increase in PAPi from 1.9 (1.3–3.0) to 3.4 (2.0–5.6; *p* = 0.002). The median duration of Impella 5.5 support was 19.0 (10.0–31.0) days, and the median time from Impella to RHC was 14 (12.0–17.0) days.

**Conclusion:**

Temporary mechanical circulatory support with the Impella 5.5 device led to rapid and significant reduction in PVR, mPAP and PASP in patients being bridged to OHT. These findings support the use of the Impella 5.5 for optimizing pre-transplant hemodynamics and suggest a potential role for tMCS in improving transplant candidacy among patients previously excluded due to PH.

## Introduction

Despite advances in guideline-directed medical therapy (GDMT), approximately 5% of the 6.2 million Americans living with heart failure (HF) progress to end-stage HF, or AHA Stage D disease, each year (1–3). While cardiac transplantation may be considered for those with advanced HF, recipients may be limited by exclusionary criteria, including pulmonary hypertension (PH), as well as by donor supply.

Pulmonary hypertension is a critically relevant comorbidity as irreversible disease limits transplant candidacy due to the risk of donor right ventricular (RV) failure in hearts unconditioned to elevated pulmonary afterload (4). Studies estimate that PH, defined as mPAP > 20mmHg, affects upwards of 60%–70% of patients with heart failure with reduced ejection fraction (HFrEF) (5). Per the 2024 International Society of Heart and Lung Transplant (ISHLT) Guidelines, a PVR >3 Wood Units (WU) warrants a stepwise evaluation of its reversibility (6). This approach typically begins with an acute vasodilator challenge, followed by hospitalization with continuous hemodynamic monitoring to assess response to medical therapy, including diuretics, inotropes, and/or vasodilators. For PH refractory to these therapies, the guidelines provide a Class 2a recommendation to consider temporary or durable mechanical circulatory support (MCS) to further assess the reversibility of PH. Notably, the evidence supporting this recommendation draws exclusively from durable MCS research, with no data from temporary devices (7–15).

First commercially available in 2019, the Impella 5.5 is a micro-axial, transaortic, temporary left ventricular support pump that reduces ventricular filling pressures and myocardial oxygen consumption while improving coronary and systemic perfusion (16). Use of the Impella 5.5 has evolved beyond treatment of acute cardiogenic shock to longer duration support as a bridge to definitive advanced HF therapy (17). Current evidence on use of temporary mechanical circulatory support (tMCS) in PH is limited to case series and small cohorts (18–22). This data suggests that the Impella 5.5 can favorably influence pulmonary hemodynamics in patients with PH from left heart disease. Broader implications for tMCS in the pretransplant PH setting remain hypothesis-generating, highlighting the need for more systematic research.

This study examined the impact of the Impella 5.5 on PH–related hemodynamic markers in patients bridged to transplant with the goal of identifying a strategy to optimize pre-transplant hemodynamics.

## Methods

### Study design

We conducted a retrospective, observational study to evaluate the effects of tMCS with the Impella 5.5 on PH related hemodynamic markers in patients undergoing OHT. This design was chosen to capture serial hemodynamic and echocardiographic data obtained before device implantation and during Impella support.

This study was conducted at two tertiary care centers with established advanced HF and cardiac transplant programs. Eligible hospitalizations occurred between October 2018 and August 2025. All data were extracted from the electronic health records (EHR) of the index admission during which an Impella 5.5 was placed, and the patient subsequently underwent OHT.

IRB approval was obtained and STROBE guidelines were followed in reporting of this observational study.

### Study patients

Patients were eligible for inclusion if they were hospitalized with HF, required tMCS with an Impella 5.5 device, and underwent orthotopic heart transplantation (OHT) during the same hospitalization, regardless of the time interval between device implantation and transplantation. In addition, patients were required to have undergone at least one right heart catheterization (RHC) or one transthoracic echocardiogram (TTE) prior to Impella placement and while the device was in place. All patients who were listed for transplant and received an Impella 5.5 were screened, even if they ultimately went on to receive LVAD or passed away in which case they were excluded. Please see Figure 1 for the study's population flow diagram.

**Figure 1:**
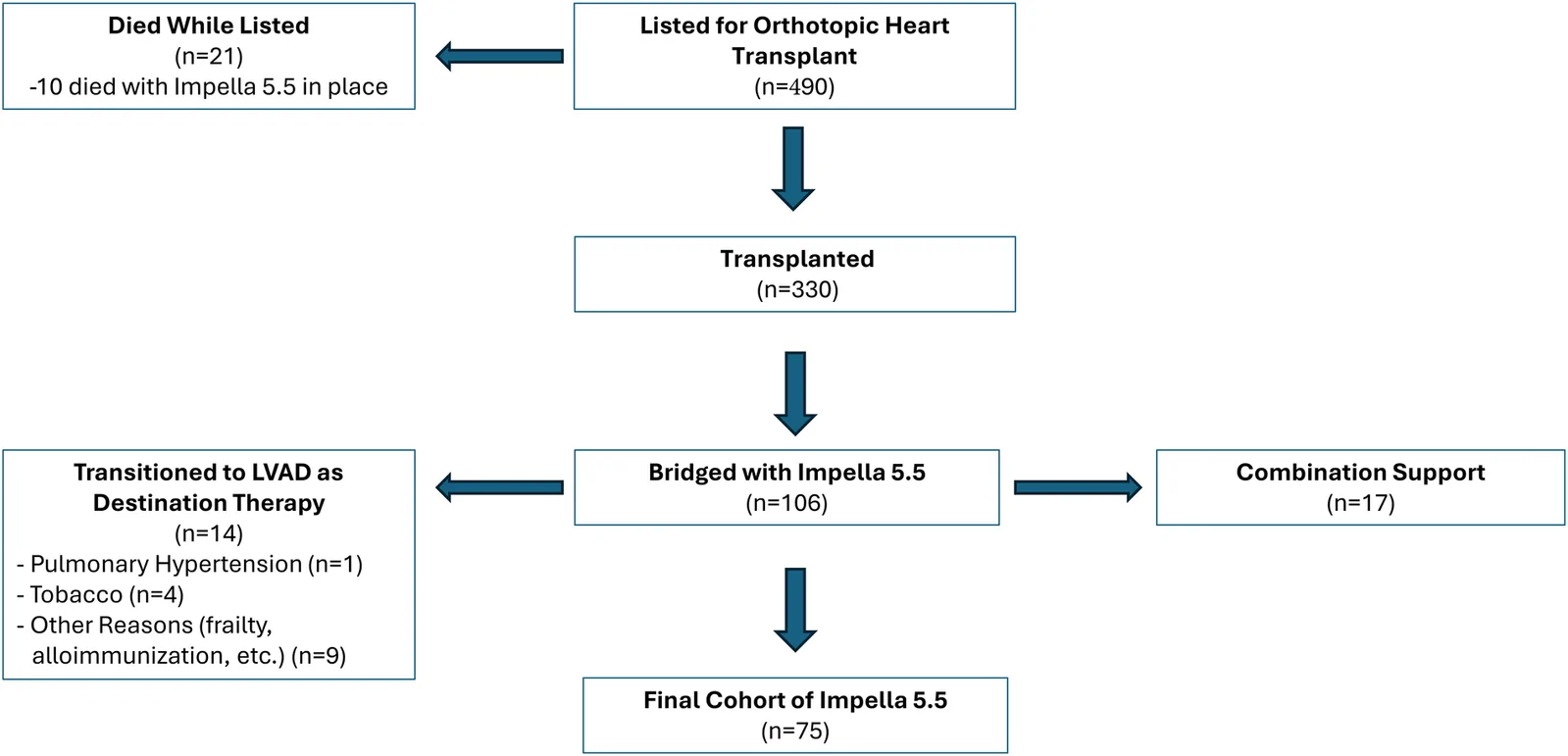
Population flow diagram.

Patients were excluded if they were cannulated by any additional mechanical circulatory support (such as extracorporeal membrane support or RV support), lacked both hemodynamic and echocardiographic data, or had incomplete records preventing outcome assessment. From the initial transplant cohort, 17 patients were excluded due to the need for ECMO support.

All eligible patients who met inclusion criteria during the study were identified through institutional procedural and transplant databases. Patient charts were reviewed to confirm study eligibility. No sample size calculation was performed because the study included all eligible cases within the designated time periods.

Data were collected from the electronic health record. RHCs were categorized into two time periods: pre-Impella (defined as all RHCs performed between hospital admission and device implantation) and during-Impella (defined as all RHCs performed while the device remained in place). TTEs were similarly grouped as pre-Impella and during Impella support. Given center-specific differences in hemodynamic assessments and data missingness, only the single closest RHC and TTE to Impella implant were used for the pre-Impella and during-Impella periods.

### Interventions

No study-specific interventions were performed. The Impella 5.5 was implanted as part of routine clinical management for cardiogenic shock or advanced HF requiring temporary LV support, including those with concomitant PH. Device insertion specifications (cannulation approach, performance level, and support duration) were determined by treating clinical teams according to institutional protocols. Hemodynamic evaluations and echocardiograms were obtained as part of standard transplant evaluation and Impella management.

### Outcomes

Primary outcomes included changes in pulmonary vascular resistance (PVR), calculated by both the Fick and thermodilution methods, mean pulmonary artery pressure (mPAP), and pulmonary artery systolic pressure (PASP). For each primary outcome, measurements obtained prior to Impella implantation were compared with values obtained while the device was in place. Secondary outcomes included qualitative right ventricular (RV) systolic function assessed by TTE, transpulmonary gradient (TPG), and pulmonary artery pulsatility index (PAPi). Additional descriptive measures included the duration of Impella support, time between Impella placement and first RHC, as well as vital signs and laboratory values obtained on the days of serial RHCs. Variables were assessed for distributional characteristics and were reported as medians with interquartile ranges.

### Statistical analysis

Continuous variables were summarized as median and interquartile range (IQR), and categorical variables were summarized as frequency and percentage. For the primary analysis, differences between the pre-Impella and during-Impella time points were assessed using the Wilcoxon rank-sum test for continuous variables and chi-square or Fisher's exact test, as appropriate, for categorical variables. The primary analysis was unpaired and included all available observations at each time point.

Additionally, paired comparisons were performed among patients with complete data at both the pre-Impella and during-Impella time points. Continuous variables were compared using the Wilcoxon signed-rank test, while categorical variables were compared using McNemar's chi-square test. For continuous variables, the paired median difference was calculated as the median of the within-patient differences between the pre-Impella and during-Impella measurements. Corresponding 95% confidence intervals (CIs) and *p*-values were reported. The number of complete patient pairs included in each paired analysis was reported as N (pairs).

A two-sided *p*-value <0.05 was considered statistically significant. Missing data were handled using pairwise deletion, such that only observations with available data for the specific variable and comparison were included in each analysis. The number of missing observations for each variable is reported in the corresponding results tables.

All statistical analyses were performed using R/RStudio version 4.4.1 with the “finalfit” and “dplyr” packages.

## Results

### Patient demographics and comorbidities

A total of 75 participants were analyzed, including 19 women and 56 men with a mean age of 56.3 ± 10.5 years. Black patients made up 42.7% of the population, and 41.3% of the population had a BMI >30 kg/m^2^. Relevant medical history included 45.3% with a history of diabetes mellitus, 49.3% with coronary artery disease, and 60% with a history of CKD3. The median EF prior to admission was 19% (IQR, 15.0- 25.0). The majority (85%) presented as NYHA Class IV. Prior to cannulation, 96% of patients were on at least one vasoactive agent, and 84% were on milrinone. The remainder of baseline demographics, comorbidities, and clinical characteristics are summarized in Table 1.

**Table 1:** Demographics, comorbidities, admission characteristics (total *N* = 75).

Demographic		Value (% or CI)
Age, years		*56.3 [45.8 to 66]*
Sex		
Female		*19* (*25.3)*
Male		*56* (*74.7)*
Race		
White		*40* (*53.3)*
Black or African American		*32* (*42.7)*
Asian		*2* (*2.7)*
Other		*1* (*1.3)*
Ethnicity		
Hispanic or Latino		*1* (*1.3)*
Not Hispanic or Latino		*73* (*97.3)*
Other		*1* (*1.3)*
Comorbidities Prior to Admission (Total *N* = 75)		**N (%)**
Anemia		*36* (*48.0)*
Atrial fibrillation or flutter		*39* (*52.0)*
Cerebrovascular accident or transient ischemic attack		*18* (*24.0)*
Chronic kidney disease stage 3a or 3b		*45* (*60.0)*
Chronic obstructive pulmonary disease		*11* (*14.7)*
Coronary artery disease		*37* (*49.3)*
Diabetes mellitus		*34* (*45.3)*
Dyslipidemia		*62* (*81.3)*
End-stage renal disease on dialysis		*2* (*2.7)*
Hypertension		*59* (*78.7)*
Peripheral arterial disease		*6* (*8.0)*
PPM/ICD/CRT		*50* (*66.7)*
Prior coronary artery bypass grafting		*10* (*13.3)*
Prior percutaneous coronary intervention		*21* (*28.0)*
Prior transcatheter valve intervention*		*5* (*6.7)*

Clinical Characteristic (*N* = 75)	**Missing (N)**	**Value (% or CI)**
Body mass index category		
Obese (≥30 kg/m**^2^**)		*31* (*41.3)*
Non-obese (<30 kg/m**^2^**)		*44* (*58.7)*
Admission Labs		
Initial Hemoglobin (g/dL)	3	*12.8 [11.7 to 14.0]*
Initial Creatinine (mg/dL)	4	*1.3 [1.0 to 1.7]*
Transthoracic Echocardiogram Data		
Ejection Fraction	1	*19.0 [15.0 to 25.0]*
LVIDD or LVEDD (mm)	6	*65.0 [57.0 to 72.0]*
Right Ventricular Dysfunction 1		
None		*29* (*39.2)*
Trace		*0* (*0.0)*
Mild		*19* (*25.7)*
Moderate		*20* (*27.0)*
Severe		*6* (*8.1)*
Aortic Regurgitation	1	
Moderate		*3* (*4.1)*
Severe		*1* (*1.4)*
Aortic Stenosis	1	
Moderate		*0* (*0.0)*
Severe		*0* (*0.0)*
Mitral Regurgitation	1	
Moderate		*29* (*39.2)*
Severe		*23* (*31.1)*
Mitral Stenosis	1	
Moderate		*0* (*0.0)*
Severe		*0* (*0.0)*
Tricuspid Regurgitation	2	
Moderate		*24* (*32.9)*
Severe		*12* (*16.4)*
Admission Characteristic (*N* = 75)		**N (%)**
Acute Heart Failure		73 (97.3)
Acute Coronary Syndrome		*2* (*2.7)*
STEMI		2 (2.7)
NYHA		
*Class I*		*0* (*0)*
*Class II*		*0* (*0)*
*Class III*		*11* (*14.6)*
*Class IV*		*64* (*85.3)*
Home Medication		
ACE inhibitor, ARB, or ARNI		*46* (*61.3)*
Beta Blocker		*37* (*49.3)*
Digoxin		*6* (*6.7)*
Loop Diuretics		*60* (*80.0)*
Mineralocorticoid Antagonist		*45* (*60.0)*
SGLT2 inhibitor		*36* (*48.0)*
Vasoactive Support Prior to Impella		
Milrinone		*63* (*84)*
Dobutamine		*8* (*10.7)*
Epinephrine		*13* (*17.3)*
Nitroprusside		*9* (*12)*
Nitric Oxide		*1* (*1.3)*
Degree of Vasoactive Support		
Zero Agents		*3* (*4)*
One Agent		*54* (*72)*
Two Agents		*16* (*21.3)*
Three Agents		*2* (*2.7)*

ACEi, angiotensin-converting enzyme inhibitor, ARB, angiotensin II receptor blocker, ARNI, angiotensin receptor-neprilysin inhibitor, CRT, cardiac resynchronization therapy, ICD, implantable cardiac defibrillator, LVEDD, left ventricular end diastolic diameter, LVIDD, left ventricular internal diameter in diastole, NYHA, New York Heart Association, PPM, permanent pacemaker, SGLT2, sodium glucose transport 2, STEMI, ST-elevation myocardial infarction.

### Primary outcomes

Following Impella 5.5 placement, median PVR by Fick and Thermodilution decreased from 2.8 (IQR, 2.1–3.8) to 1.5 (1.2–2.1) Wood Units (WU) (*p* < 0.001) and from 2.4 (1.9–2.8) to 1.3 (1.0–1.5) WU (*p* < 0.001), respectively. Median mPAP and PASP decreased from 35.5 (31.0–41.8) to 24.5 (20.2–32.0) mmHg (*p* < 0.001) and from 48 (42.0–55.8) to 37.5 (27.5–42.0) mmHg (*p* < 0.001), respectively. All unpaired outcomes and additional hemodynamic and echocardiographic data can be found in Table 2. Primary and secondary outcomes are also reflected in Figure 2.

**Table 2:** Outcomes.

Hemodynamic Measure	Missing Pre- or During-(N)	Pre-Impella	During-Impella	*P*-Value
Total *N* = 150		*N* = 75	*N* = 75	
Vitals				
Mean Arterial Pressure (mmHg)	46	88.0 [82.0 to 96.0]	87.0 [81.2 to 94.0]	0.549
Systolic Blood Pressure (mmHg)	46	111.0 [100.5 to 119.8]	105.5 [99.2 to 120.0]	0.240
Heart Rate (bpm)	66	92.5 [81.2 to 106.0]	95.5 [84.5 to 102.0]	0.645
Hemodynamics				
Pulmonary Artery Pulsatility Index	49	1.9 [1.3 to 3.0]	3.4 [2.0 to 5.6]	**0**.**002**
Mean Pulmonary Artery Pressure (mmHg)	46	35.5 [31.0 to 41.8]	24.5 [20.2 to 32.0]	**<0**.**001**
Pulmonary Artery Systolic Pressure (mmHg)	46	48 [42.0 to 55.8]	37.5 [27.5 to 42.0]	**<0**.**001**
Pulmonary Artery Diastolic Pressure (mmHg)	46	25.0 [ 21.2 to 31.8]	16.0 [13.2 to 23.0]	**<0**.**001**
Transpulmonary Gradient (TPG) (mmHg)	50	9.5 [7.0 to 12.0]	8.0 [6.8 to 11.0]	**0**.**356**
Pulmonary Capillary Wedge Pressure (mmHg)	46	26.0 [20.0 to 31.0]	18.0 [13.0 to 21.8]	**<0**.**001**
PVR Fick (Wood Units)	52	2.8 [2.1 to 3.8]	1.5 [1.2 to 2.1]	**<0**.**001**
PVR Thermodilution (Wood Units)	125	2.4 [1.9 to 2.8]	1.3 [1.0 to 1.5[	**<0**.**001**
Cardiac Index Fick (L/min/m^2**)**^	49	1.7 [1.5 to 1.9]	2.5 [2.2 to 2.9]	**<0**.**001**
Cardiac Index Thermo (L/min/m^2^)	124	1.8 [1.7 to 2.4]	2.7 [2.3 to 2.9]	**0**.**003**
Cardiac Output Fick (L/min)	53	3.6 [3.1 to 4.1]	4.9 [4.1 to 5.7]	**<0**.**001**
Cardiac Output Thermo (L/min)	125	3.6 [3.2 to 5.0]	5.3 [4.5 to 6.0]	**0**.**017**
Right Atrial Pressure (mmHg)	47	11.5 [8.0 to 16.0]	6.0 [4.0 to 9.0]	**<0**.**001**
Days Between Impella and RHC		–	14.0 [12.0 to 17.0]	–
Duration of Impella Support (days)			19.0 [10.0 to 31.0]	
TTE Data				
Ejection Fraction	*10*	*17.5 [15.0 to 20.8]*	*15 [13.1 to 17.5]*	***0***.***017***
LVIDD or LVEDD	*34*	*65.0 [59.0 to 71.5]*	*62.0 [57.0 to 68.0]*	*0*.*075*
RV Dysfunction	*13*			
Moderate to Severe		*27 (36.5)*	*22 (34.9)*	0.865
Aortic Regurgitation	45			
None to Mild		68 (94.4)	33 (100.0)	0.311
Moderate to Severe		*4 (82.5)*	0	
Mitral Stenosis	56			
None to Mild		*72 (100)*	*21 (95.5)*	*0*.*237*
Moderate to Severe		*0*	*1 (4.5)*	
Mitral Regurgitation	21			
None to Mild		*20 (27)*	*26 (47.3)*	***0***.***021***
Moderate to Severe		*54 (73)*	*29 (52.7)*	
Tricuspid Regurgitation	32			
None to Mild		*38 (52.1)*	*28 (62.2)*	0.329
Moderate to Severe		*35 (47.9)*	*17 (37.8)*	

**Figure 2:**
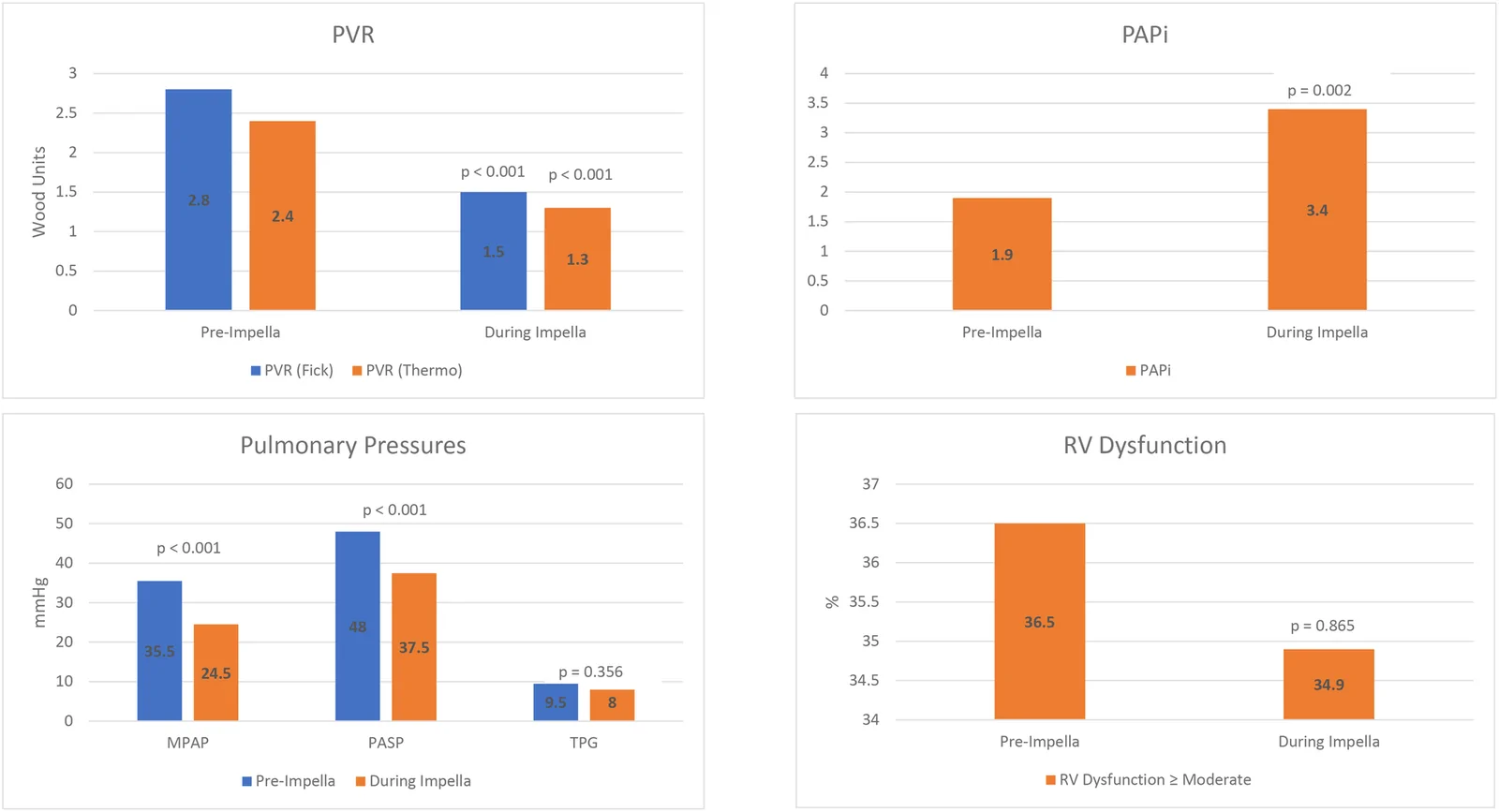
Primary and secondary outcome measures pre and during impella including: PVR by fick and thermodilution, mPAP, PASP, TPG, PAPi and ≥ moderate RV dysfunction.

Paired analysis was conducted for those patients with both pre-Impella and during Impella hemodynamic data and revealed similar results in our primary outcomes. Specific data can be found in Supplementary Table S1.

### Secondary outcomes

Impella 5.5 implantation was associated with a significant increase in PAPi after Impella placement, from 1.9 (1.3–3.0) to 3.4 (2.0–5.6; *p* = 0.002). There was no statistically significant difference in TPG or the proportion of patients with at least moderate qualitative RV dysfunction following Impella placement. The median time from Impella implantation to the first RHC was 14.0 days (12.0–17.0). The median duration of Impella 5.5 support was 19.0 days (10.0–31.0).

### Severe PH subgroup analysis

A paired analysis of patients with a baseline PVR by Fick or Thermodilution >3 WU (*n* = 27) was completed and showed a reduction in PVR by Fick from 4.0 to 2.0 WU (paired median difference, −2.39 WU; 95% CI, −3.42 to −1.36; *p* = 0.002). mPAP decreased from 42.0 to 29.0 mmHg (−11.7 mmHg; 95% CI, −20.5 to −3; *p* = 0.016) and PASP decreased from 54.0 to 40.0 mmHg (−14.75 mmHg; 95% CI, −25.5 to −5; *p* = 0.011). Regarding secondary outcomes, TPG decreased significantly from 13.5 to 9.0 mmHg (−5.5 mmHg; 95% CI, −7.5 to −2.5; *p* = 0.012).

Of the patients who had an Impella 5.5 placed and had a transplant evaluation, only one was ultimately declined for transplant because of severe PH. The patient did not undergo RHC with Impella in place thus limiting assessment of its hemodynamic effects.

### Laboratory data

Laboratory parameters were assessed before and during Impella 5.5 support on days when right heart catheterization was performed (Table 3) with statistically significant reduction in lactate following placement of Impella 5.5 as well as a decrease in hemoglobin.

**Table 3:** Laboratory parameter data.

Laboratory Parameter	Missing Pre- or During- (N)	Pre-Impella	During-Impella	*P*-Value
Total *N* = 150		*N* = 75	*N* = 75	
BNP (pg/mL)	*122*	*1004.0 [578.0 to 1744.0]*	*302.0 [292.0 to 433.5]*	*0*.*058*
Creatinine (mg/dL)	*49*	*1.2 [1.0 to 1.7]*	*1.2 [0.9 to 1.4]*	*0*.*184*
eGFR (mL/min/1.73m^2^)	*50*	*59.0 [45.0 to 78.5]*	*65.0 [51.0 to 89.0]*	*0*.*260*
Albumin (g/dL)	*82*	*3.7 [3.6 to 4.0]*	*3.6 [3.3 to 3.7]*	*0*.*093*
Lactate (mmol/L)	*118*	*1.3 [0.8 to 1.8]*	*0.8 [0.7 to 1.0]*	***0***.***048***
LDH (U/L)	*114*	*252.0 [177.5 to 288.0]*	*315.0 [259.0 to 422.0]*	*0*.*054*
Haptoglobin (mg/dL)	*145*	*115.0 [111.0 to 139.0]*	*30.0 [30.0 to 30.0]*	*0*.*076*
Total Bilirubin (mg/dL)	*80*	*1.2 [0.7 to 1.8]*	*0.9 [0.7 to 1.1]*	*0*.*089*
Hemoglobin (g/dL)	*50*	*13.0 [11.7 to 14.3]*	*9.9 [8.6 to 11.0]*	***<0***.***001***

Paired analysis of these variables revealed an increase in eGFR from 59.0 to 65.0 mL/min/1.73m^2^ (7.0 mL/min/1.73m^2^; 95% CI, 0–15.0; *p* = 0.045). Total bilirubin decreased from 1.2 to 0.9 mg/dL (−0.58 mg/dL; 95% CI, −1 to −0.15; *p* = 0.018) and hemoglobin decreased significantly from 13.0 to 9.0 g/dL (−2.95 g/dL; 95% CL, −3.6 to −2.25; *p* < 0.001) (Supplementary Table S2).

Paired analysis of standing weight on day of pre-Impella RHC compared to weight on the day of during- Impella RHC decreased from 88.4 to 84.5 kg (−2.4 kg; 95% CI, −5.3 to −0.2; *p* = 0.033).

### Clinical outcomes and safety data/complications

No deaths occurred during the index admission or within 30 days of discharge. Readmission within 30 days occurred in 9 patients (12%) (Table 4).

**Table 4:** Clinical outcome measures (*N* = 75).

Clinical Outcome Measure	Value
Mortality	
30 Day	*0* (*0)*
In-hospital	*0* (*0)*
Length of Stay (days)	*45.91*
All-Cause Rehospitalization within 30 days of Discharge	*9* (*12.0)*
Stroke	*3* (*4.0)*
Composite Safety Endpoint	
Hemolysis	*12* (*16.0)*
Hgb drop >5 g/dL	*13* (*17.3)*
Operative Device Complications	*6* (*8.0)*
Limb Ischemia	*2* (*2.7)*
Need for Hemodialysis	*3* (*4.0)*

Complications during Impella use included the following: 1) hemolysis, defined as LDH ≥ 870 U/L (3x upper limit of normal), occurred in 12 patients (16%) (managed with a reduction in Impella performance level when feasible); 2) hemoglobin dro*p* ≥ 5 g/dL, including in the perioperative period, occurred in 13 patients (17%); 3) device complications, defined as device related hematoma or malposition requiring operative intervention, occurred in 6 patients (8.0%); 4) device-related limb ischemia, occurred in 2 patients (3%) both of whom developed right upper extremity ischemia related to embolic phenomenon immediately following device removal and required surgical intervention; 5) need for hemodialysis, occurred in 3 patients (4%), all of which occurred after transplant; 6) acute stroke, occurred in 3 patients (4%).

## Discussion

Pre-transplant PH remains a significant barrier to OHT, as it is a well-established independent predictor of post-transplant morbidity and mortality driven largely by the risk of RV failure. In this study, we demonstrate that temporary LV unloading with the Impella 5.5 results in rapid and clinically meaningful improvements in PVR and pulmonary hemodynamics in patients with advanced HF and concomitant PH. In doing so, we highlight the role for short-term tMCS in optimizing transplant candidacy.

The existing evidence linking LV unloading to improvements in PH is almost entirely derived from observational studies in the durable LVAD population with limited evidence regarding the role of tMCS (14). To date, several small studies have examined the effect of tMCS on pulmonary hemodynamics (18–21). Our study confirms their preliminary observations while providing a larger, more rigorously characterized cohort with comprehensive serial invasive hemodynamic, echocardiographic, and safety data that were not available in the earlier abstracts. Our data aligns with prior LVAD and tMCS trends, showing significant reductions in PVR and pulmonary pressures.

Several features of our study support the validity of these findings. We observed consistent improvements across multiple complementary domains, including invasive pulmonary hemodynamics, surrogate measures of RV performance, and laboratory markers of end-organ dysfunction and congestion. The concordance of these parameters strengthens the likelihood that the observed changes reflect true physiologic improvement rather than measurement variability or regression to the mean. Although not all parameters reached statistical significance, the direction of change consistently favored improvement with Impella, suggesting that the absence of significance likely reflects limited statistical power. Furthermore, the direction of these changes aligns with previously published data, supporting reproducibility across centers and patient populations. Device-related complications with Impella 5.5 were consistent with prior reports with comparable rates of hemolysis, reduction in hemoglobin, limb ischemia, and infrequent need for operative intervention (16, 22–29). The ischemic stroke rate was 4%, similar to ranges cited by prior publications (16, 23–25, 27–29).

Our data is further supported by logical and studied physiologic mechanisms. Pulmonary vascular remodeling in left heart disease begins with chronically elevated left ventricular (LV) filling pressures and transmission into the pulmonary venous circulation, creating a state of post-capillary PH. Over time, sustained pressures and endothelial stress trigger structural changes in the pulmonary vasculature, including smooth muscle cell proliferation, medial hypertrophy, and intimal thickening (30–32). These changes narrow the vascular lumen and increase pulmonary vascular resistance, resulting in an evolution to a mixed pre- and post-capillary hypertension that can often be refractory to pharmacotherapy. Unloading the left ventricle, whether with durable LVADs or temporary devices like Impella 5.5, removes the stimulus for endothelial stress. With sustained unloading over time, reverse remodeling of the pulmonary vasculature may occur, manifesting as reductions in PVR and structural reversal of medial hypertrophy and intimal changes as seen in animal models by Tian et al. (33) While our data cannot directly delineate the relative contributions of these mechanisms, they are supported by physiologic principles and prior observations in related populations. Notably, TPG decreased following Impella placement but did not do so significantly in our primary analysis. This finding may suggest that the observed hemodynamic improvements were primarily driven by LV unloading rather than true reversal of pulmonary vascular remodeling, which would be expected to reduce TPG. In a subgroup analysis of a small number of patients with more severe PH (>3 WU), there was a significant reduction in TPG (Supplementary Table S3). Given these findings, larger and longer support duration studies are warranted to determine whether tMCS can meaningfully reverse pre-capillary or “fixed” PH as has been seen in LVAD studies (7, 34).

Our findings have important clinical implications for the management of advanced HF patients with PH who are being evaluated for transplant. Current ISHLT guidance regarding the use of tMCS to assess reversibility of PH is largely extrapolated from durable LVAD experience, raising concerns about its applicability in contemporary bridge-to-transplant strategies. Our study supports a potential role for tMCS, specifically the Impella 5.5, early in the pretransplant population to actively optimize pulmonary vascular hemodynamics.

This study has several strengths, including its two-center design and integrated assessment of invasive hemodynamics, echocardiography, and clinically meaningful laboratory outcomes. However, several limitations exist. The retrospective design and modest sample size limited causal inference, and differences in right heart catheterization practices between centers introduced variability in longitudinal hemodynamic assessment and a significant degree of data missingness. Specifically, abbreviated RHC protocols were used at one site with the Impella in place, thus limiting hemodynamic data for these patients and resulting in a lower number of data points while the Impella was in place. Notably, despite the small numbers included, a paired analysis of those with complete data sets revealed consistent improvements in all primary and some secondary outcomes. Confounding from concomitant HF therapies (inotropes, diuretics, vasodilators) was inherent to the observational study design and could not be fully controlled. Standing weight on the days of RHC demonstrated a statistically significant reduction, suggesting improved volume status. Although volume reduction could theoretically confound the observed improvement in pulmonary hemodynamics, we believe diuresis and MCS are inherently interrelated, as Impella 5.5 support augments forward flow and may improve renal perfusion and function, thereby facilitating effective diuresis. Accordingly, the observed weight reduction may represent a downstream effect of MCS rather than an independent confounder. Additionally, the absence of a non-MCS control group limits comparative interpretation, though withholding temporary MCS in this high-risk population would be unethical.

Finally, selection bias likely influenced which patients received Impella 5.5 support, potentially excluding individuals with severe pre-capillary or mixed PH. Importantly, we reviewed those patients with Impella 5.5 with open evaluation who did not progress to advanced therapies or underwent LVAD placement. Of these, only one patient was excluded from OHT due to irreversible PH. Unfortunately, this patient did not undergo repeat RHC while supported with Impella, limiting assessment of hemodynamic response. A subgroup analysis was performed among patients with a baseline PVR >3 WU to evaluate those with more severe PH. Despite the small sample size, this subgroup demonstrated significant improvements in our primary outcomes as well as TPG. Notably, the median PVR decreased from 4.0 WU to 2.0 WU, suggesting that Impella implantation may allow patients with initially unfavorable hemodynamics to achieve parameters considered acceptable for transplantation. Given the limitations of this analysis, generalizability is restricted, and larger prospective studies with standardized hemodynamic monitoring protocols are needed to better define the extent to which temporary tMCS can improve pulmonary hemodynamics in patients with significant PH.

## Conclusion

In conclusion, our study presents a comprehensive evaluation of Impella 5.5–mediated LV unloading and its effects on PH in the pre-transplant population. The consistent and significant improvements in pulmonary hemodynamics observed in our cohort reinforce the concept that temporary LV unloading can meaningfully lower pulmonary pressures and PVR, supporting a role during transplant evaluation for patients with unfavorable hemodynamics. Future prospective studies assessing larger cohorts of patients with more severe, or fixed PH, and their response to tMCS are warranted.

## Data Availability

The raw data supporting the conclusions of this article will be made available by the authors, without undue reservation.
