# Supplementary material for: Improvement in pulmonary vascular resistance with IMPELLA 5.5 as a bridge to orthotopic heart transplantation
Source: Front Cardiovasc Med. 2026 Oct 20;13:1935715. doi: 10.3389/fcvm.2026.1935715 (PMC13727625; doi:10.3389/fcvm.2026.1935715)
Supplement: Supplementary file 1 [file Table1.pdf]

Supplementary Table S1: Paired Analysis of Outcomes

Hemodynamic Measure	Total N	Missing N	levels	Pre-Impella	During-Impella	Total	Paired Median Diff	95% CI	N (pairs)	P-value
Total N (%)				75 (50.0)	75 (50.0)	150				
Mean Arterial Pressure (mmHg)	104 (69.3)	46	Median (IQR)	88.0 (82.0 to 96.0)	87.0 (81.2 to 94.0)	87.5 (82.0 to 95.2)	-3.5	-9.5 to 2.5	33	0.144
Systolic BP (mmHg)	104 (69.3)	46	Median (IQR)	111.0 (100.5 to 119.8)	105.5 (99.2 to 120.0)	109.0 (100.0 to 120.2)	-8	-13.5 to -0.5	33	**0.041**
HR	84 (56.0)	66	Median (IQR)	92.5 (81.2 to 106.0)	95.5 (84.5 to 102.0)	94.0 (83.0 to 105.0)	5.5	-1 to 13	26	0.106
Bed Weight (Kg)	18 (12.0)	132	Median (IQR)	83.6 (75.0 to 93.2)	74.0 (67.9 to 96.9)	80.3 (72.6 to 96.0)	-1.35	-3.9 to 1.2	2	1
Standing Weight (Kg)	80 (53.3)	70	Median (IQR)	88.4 (81.0 to 98.2)	84.5 (79.6 to 101.8)	87.3 (80.4 to 100.1)	-2.4	-5.3 to -0.2	20	**0.033**
PAPi	101 (67.3)	49	Median (IQR)	1.9 (1.3 to 3.0)	3.4 (2.0 to 5.6)	2.3 (1.5 to 3.5)	1.44	0.45 to 2.68	30	**0.009**
TPG (mmHg)	100 (66.7)	50	Median (IQR)	9.5 (7.0 to 12.0)	8.0 (6.8 to 11.0)	9.0 (7.0 to 12.0)	-1	-2.5 to 1	29	0.308
mPAP (mmHg)	104 (69.3)	46	Median (IQR)	35.5 (31.0 to 41.8)	24.5 (20.2 to 32.0)	32.5 (24.0 to 38.0)	-10.5	-15 to -6	33	**<0.001**
PASP (mmHg)	104 (69.3)	46	Median (IQR)	48.0 (42.0 to 55.8)	37.5 (27.5 to 42.0)	44.0 (35.0 to 53.0)	-13	-19 to -7.5	33	**<0.001**
PADP (mmHg)	104 (69.3)	46	Median (IQR)	25.0 (21.2 to 31.8)	16.0 (13.2 to 23.0)	24.0 (16.0 to 30.0)	-7.5	-11.5 to -4	33	**<0.001**
PCWP (mmHg)	104 (69.3)	46	Median (IQR)	26.0 (20.0 to 31.0)	18.0 (13.0 to 21.8)	22.0 (17.0 to 29.0)	-9	-14 to -4.5	33	**0.001**
PVR Fick (WU)	98 (65.3)	52	Median (IQR)	2.8 (2.1 to 3.8)	1.5 (1.2 to 2.1)	2.4 (1.5 to 3.2)	-1.16	-1.74 to -0.6	30	**<0.001**
PVR Thermo (WU)	25 (16.7)	125	Median (IQR)	2.4 (1.9 to 2.8)	1.3 (1.0 to 1.5)	1.8 (1.4 to 2.4)	-1.28	-1.93 to -0.5	9	**0.008**
CI Fick (L/min/m^2)	101 (67.3)	49	Median (IQR)	1.7 (1.5 to 1.9)	2.5 (2.2 to 2.9)	1.8 (1.6 to 2.3)	0.75	0.51 to 1	31	**<0.001**
CI Thermo (L/min/m2)	26 (17.3)	124	Median (IQR)	1.8 (1.7 to 2.4)	2.7 (2.3 to 2.9)	2.3 (1.7 to 2.7)	0.57	0.03 to 1.5	10	**0.049**
CO Fick (L/min)	97 (64.7)	53	Median (IQR)	3.6 (3.1 to 4.1)	4.9 (4.1 to 5.7)	3.8 (3.4 to 4.7)	1.24	0.76 to 1.88	28	**<0.001**
CO Thermo (L/min)	25 (16.7)	125	Median (IQR)	3.6 (3.2 to 5.0)	5.3 (4.5 to 6.0)	4.5 (3.4 to 5.3)	0.85	-0.33 to 3.2(	9	0.203
Right Atrial Pressure (mmHg)	103 (68.7)	47	Median (IQR)	11.5 (8.0 to 16.0)	6.0 (4.0 to 9.0)	10.0 (5.0 to 15.0)	-5	-8 to -2	32	**0.002**
Days Between Impella and RHC					14.0 (12.0 to 17.0)	14.0 (12.0 to 17.0)				
**TTE Data**										
Ejection Fraction (%)	140 (93.3)	10	Median (IQR)	17.5 (15.0 to 20.8)	15.0 (13.1 to 17.5)	16.5 (15.0 to 20.0)	-3	-5 to -1.25	65	**0.002**
LVIDD or LVEDD (mm)	116 (77.3)	34	Median (IQR)	65.0 (59.0 to 71.5)	62.0 (57.0 to 68.0)	64.5 (58.8 to 70.0)	-1	-3 to 1	44	0.357
RV Dysfunction	137 (91.3)	13	None to Mild	47 (63.5)	41 (65.1)	88 (64.2)	-	-	62	1
			Moderate to Severe	27 (36.5)	22 (34.9)	49 (35.8)				
Left Atrial Dilatation	90 (60.0)	60	None to Mild	28 (39.4)	11 (57.9)	39 (43.3)	-	-	18	0.077
			Moderate to Severe	43 (60.6)	8 (42.1)	51 (56.7)				
Aortic Regurgitation	105 (70.0)	45	None to Mild	68 (94.4)	33 (100.0)	101 (96.2)	-	-	30	0.48
			Moderate to Severe	4 (5.6)		4 (3.8)				
Mitral Regurgitation	129 (86.0)	21	None to Mild	20 (27.0)	26 (47.3)	46 (35.7)	-	-	54	**0.001**
			Moderate to Severe	54 (73.0)	29 (52.7)	83 (64.3)				
Mitral Stenosis	94 (62.7)	56	None to Mild	72 (100.0)	21 (95.5)	93 (98.9)	-	-	20	
			Moderate to Severe		1 (4.5)	1 (1.1)				
Tricuspid Regurgitation	118 (78.7)	32	None to Mild	38 (52.1)	28 (62.2)	66 (55.9)	-	-	44	**0.043**
			Moderate to Severe	35 (47.9)	17 (37.8)	52 (44.1)				
