# Supplementary material for: Improvement in pulmonary vascular resistance with IMPELLA 5.5 as a bridge to orthotopic heart transplantation
Source: Front Cardiovasc Med. 2026 Oct 20;13:1935715. doi: 10.3389/fcvm.2026.1935715 (PMC13727625; doi:10.3389/fcvm.2026.1935715)
Supplement: Supplementary file 2 [file Table2.pdf]

Supplementary Table S2: Paired Lab Value Analysis

Lab Value	Total N	Missing N	Levels	Pre-Impella	During Impella	Total	Paired Median Diff	95% CI	N (pairs)	P-value
Total N (%)				75 (50.0)	75 (50.0)	150				
BNP	28 (18.7)	122	Median (IQR)	1004.0 (578.0 to 1744.0)	302.0 (292.0 to 433.5)	948.0 (531.2 to 1739.5)	-683.25	-995 to -296	3	0.25
Troponin	8 (5.3)	142	Median (IQR)	22.0 (18.0 to 46.0)	16.0 (16.0 to 16.0)	21.0 (16.0 to 42.5)			1	
Creatinine	101 (67.3)	49	Median (IQR)	1.2 (1.0 to 1.7)	1.2 (0.9 to 1.4)	1.2 (0.9 to 1.6)	-0.15	-0.3 to 0	32	**0.048**
eGFR	100 (66.7)	50	Median (IQR)	59.0 (45.0 to 78.5)	65.0 (51.0 to 89.0)	62.0 (49.0 to 80.0)	7	0 to 15	31	**0.045**
Albumin	68 (45.3)	82	Median (IQR)	3.7 (3.6 to 4.0)	3.6 (3.3 to 3.7)	3.7 (3.5 to 4.0)	-0.15	-0.45 to 0.2	10	0.332
Lactate	32 (21.3)	118	Median (IQR)	1.3 (0.8 to 1.8)	0.8 (0.7 to 1.0)	1.1 (0.8 to 1.7)	-0.4	-1.23 to 0.35	7	0.219
LDH	36 (24.0)	114	Median (IQR)	252.0 (177.5 to 288.0)	315.0 (259.0 to 422.0)	275.0 (220.0 to 395.0)	46.5	-154 to 231	4	0.625
Haptoglobin	5 (3.3)	145	Median (IQR)	115.0 (111.0 to 139.0)	30.0 (30.0 to 30.0)	107.0 (30.0 to 115.0)			0	
Total Bilirubin	70 (46.7)	80	Median (IQR)	1.2 (0.7 to 1.8)	0.9 (0.7 to 1.1)	1.1 (0.7 to 1.6)	-0.58	-1 to -0.15	11	**0.018**
Hemoglobin	100 (66.7)	50	Median (IQR)	13.0 (11.7 to 14.3)	9.9 (8.6 to 11.0)	12.3 (10.4 to 13.7)	-2.95	-3.6 to -2.25	32	**<0.001**
