# Supplementary material for: Improvement in pulmonary vascular resistance with IMPELLA 5.5 as a bridge to orthotopic heart transplantation
Source: Front Cardiovasc Med. 2026 Oct 20;13:1935715. doi: 10.3389/fcvm.2026.1935715 (PMC13727625; doi:10.3389/fcvm.2026.1935715)
Supplement: Supplementary file 3 [file Table3.pdf]

Supplementary Table S3: Subgroup Analysis PVR >3 WU

Hemodynamic Measure	Total N	Missing N	Levels	Pre-Impella	During Impella	Total	Paired Median Diff	95% CI	N (pairs)	P-value
Total N (%)				27 (50.0)	27 (50.0)	54				
MAP	38 (70.4)	16	Median (IQR)	88.0 (82.5 to 98.2)	92.5 (85.0 to 96.0)	90.0 (82.5 to 98.2)	2.26	-6 to 13	12	0.722
Systolic BP	38 (70.4)	16	Median (IQR)	112.5 (106.2 to 121.0)	113.5 (102.8 to 125.2)	112.5 (103.5 to 121.8)	0.57	-11 to 15.5	12	1
HR	25 (46.3)	29	Median (IQR)	97.0 (85.8 to 108.5)	95.0 (93.5 to 97.0)	95.0 (88.0 to 103.0)	0.5	-14 to 12.5	7	1
Bed Weight	7 (13.0)	47	Median (IQR)	83.6 (77.0 to 93.2)	78.6 (73.2 to 83.9)	83.6 (76.0 to 91.2)			1	
Standing Weight	27 (50.0)	27	Median (IQR)	84.2 (79.7 to 91.2)	82.8 (76.6 to 111.0)	83.7 (78.0 to 93.3)	-1.65	-5.3 to 2	6	0.498
PAPi	38 (70.4)	16	Median (IQR)	1.8 (1.6 to 2.7)	3.9 (2.0 to 5.4)	2.1 (1.6 to 4.0)	1.11	-1.01 to 3.23	11	0.221
TPG	37 (68.5)	17	Median (IQR)	13.5 (11.0 to 16.8)	9.0 (8.0 to 12.0)	12.0 (10.0 to 15.0)	-5.5	-7.5 to -2.5	10	**0.012**
mPAP	39 (72.2)	15	Median (IQR)	42.0 (33.0 to 48.0)	29.0 (23.2 to 33.2)	37.0 (31.5 to 46.0)	-11.7	-20.5 to -3	12	**0.016**
PASP	39 (72.2)	15	Median (IQR)	54.0 (46.5 to 66.5)	40.0 (32.2 to 44.8)	49.0 (42.0 to 63.0)	-14.75	-25.5 to -5	12	**0.011**
PADP	39 (72.2)	15	Median (IQR)	30.0 (24.5 to 36.5)	19.5 (15.5 to 24.0)	27.0 (22.0 to 35.5)	-9	-19 to 0.5	12	0.056
PCWP	39 (72.2)	15	Median (IQR)	25.0 (20.0 to 35.0)	20.0 (16.8 to 23.2)	22.0 (19.0 to 31.0)	-7	-19 to 6.5	12	0.261
PVR Fick	38 (70.4)	16	Median (IQR)	4.0 (3.4 to 5.0)	2.0 (1.6 to 2.7)	3.7 (2.9 to 4.4)	-2.39	-3.42 to -1.36	11	**0.002**
PVR Thermo	8 (14.8)	46	Median (IQR)	3.2 (2.8 to 3.9)	1.3 (1.3 to 1.4)	2.8 (1.4 to 3.4)	-1.76	-2.58 to -1.19	3	0.25
CI Fick	38 (70.4)	16	Median (IQR)	1.6 (1.4 to 1.8)	2.4 (1.9 to 2.9)	1.8 (1.5 to 2.0)	0.81	0.28 to 1.37	11	**0.008**
CI Thermo	8 (14.8)	46	Median (IQR)	1.7 (1.4 to 1.7)	2.9 (2.9 to 2.9)	2.2 (1.6 to 2.9)	0.94	0.16 to 1.21	3	0.25
CO Fick	35 (64.8)	19	Median (IQR)	3.4 (3.1 to 3.6)	4.6 (3.8 to 4.9)	3.6 (3.2 to 4.1)	1.09	0.3 to 2.46	9	**0.021**
CO Thermo	7 (13.0)	47	Median (IQR)	2.8 (2.8 to 3.6)	5.8 (5.6 to 5.9)	3.6 (2.8 to 5.5)	1.2	-0.1 to 2.5	2	1
RAP	39 (72.2)	15	Median (IQR)	12.0 (8.5 to 16.5)	6.5 (4.0 to 10.5)	10.0 (5.5 to 15.0)	-5	-9.5 to 3.5	12	0.182
Days Between Impella and RHC						13.0 (12.0 to 15.0)				
